# The Effect of Illumination on Positive Fusional Vergence

**DOI:** 10.22599/bioj.296

**Published:** 2023-09-29

**Authors:** Raisul Azam, Sourav Karmakar, Animesh Mondal, Gaurav Kumar Bhardwaj

**Affiliations:** 1 Department of Optometry and Vision Science, Amity University, Gurgaon, Haryana, India

**Keywords:** positive fusional vergence, illumination, asthenopia

## Abstract

**Background:**

Positive fusional vergence (PFV) is vital in maintaining fusion in critical and continuous near tasks such as reading or performing digital screen tasks. This study investigated how PFV changed under various lighting conditions.

**Methods:**

This cross-sectional study recruited 34 participants aged between 21 and 25 years, with best corrected visual acuity (BCVA) 0.0 logMAR and insignificant refractive error. Three different illuminations—low illumination (50 lux), medium lighting (100 lux), and high illumination (150 lux)—were used to examine the ocular parameters PFV (blur, break, and recovery points), contrast sensitivity and pupil diameter.

**Results:**

Pupil diameter changed significantly in different room illuminations (*p* = 0.00). There was no significant difference in contrast sensitivity across the three levels of room illumination (*p* = 0.368). Mean PFV (SD) (blur) was 14.5 (2.5) in 50 lux, 10.2 (2.2) in 100 lux, and 8.2 (2.1) in 150 lux. Under 50, 100 and 150 lux, respectively, the mean PFV (SD) (break) values were 16.7 (2.4), 13.4 (1.8), and 10.8 (2.2), and the mean PFV (SD) (recovery) values were 13.3 (2.1), 10.7 (2.1), and 7.5 (2.7). With increased illumination levels, PFV blur, break, and recovery values were significantly lower (*p* < 0.001).

**Conclusions:**

PFV values were significantly higher in lower illumination. Clinicians should be aware that room illumination affected the PFV values measured.

## Introduction

Illumination, a crucial workplace component, impacts employees’ productivity and working conditions. Appropriate illumination degree and distribution are required for employees to operate at their best. *“Varying levels of illumination may impact the ocular function”, “Advice on the optimum standards for reading illumination has been suggested by various organisations”*, and *“The Indian National Electric Code of 2011 recommended that illumination for reading should be 300–700 lux, and the Central Building Research Institute of India advises 200–500 lux for tasks like reading”* (Ram & Bhardwaj 2018). These recommendations are based on the Indian Standard Code of Practice for Industrial Illumination (Ram & Bhardwaj 2018).

According to recent studies, the quantity and quality of the illumination may significantly influence behaviour (Lança & Rowe 2019). Adequate fusional amplitudes are required for binocular vision stability. In the presence of vergence anomalies, a wide range of symptoms may be experienced, interfering with visual comfort and academic performance (Ansons & Davis 2013). As fusional vergence is necessary for maintaining fusion, clinicians typically investigate horizontal, and at times vertical, fusional vergence ranges for distance and near vision (Ansons & Davis 2013). These measurements are not typically recorded in different illumination levels (Lança & Rowe 2019). Positive fusional vergence (PFV) measurements can be determined by the responses reported by the patient, including the blur point, break point and recovery point (Ansons & Davis 2013).

A previous study of accommodation and convergence levels using a 3D object in two different illumination levels revealed that accommodation led the convergence in brighter lighting. In contrast, accommodation and convergence were identical in average illumination (Jiang et al. 1991). In low illumination, convergence led the accommodation (Wolska 2003). Convergence and accommodation efforts have been shown to increase in low illumination levels, whereas in a brighter environment, the subject needs less accommodation and convergence (Okada et al. 2013).

Visual problems, attention issues, nutritional deficiencies, and general declines in health and mood have all been linked to inadequate illumination. It has been shown that the proper illumination can help counteract these effects, boost academic performance, and solve challenges. Brightness can significantly impact behaviour, as well as school and workplace performance (Abramson et al. 2007). Increased light brightness has been demonstrated to reduce stressful workload and boost productivity (Abramson et al. 2007).

This study measured near PFV, contrast sensitivity, and pupil size under different illumination levels to evaluate whether illumination could have an effect on these measures of visual function.

## Methods

### Participants

Ethical approval was granted for this study by the Institutional Ethics Committee for Human Research of Amity University Haryana (Ref No.- IEC -AIB/AUH/2021-22). A sample size of 34 was calculated (see Appendix). Fifty students from Amity University, Haryana, were screened for participation in this cross-sectional study. During the screening, they were provided with information about the study, and their consent was obtained. During the screening, participants underwent a measurement of visual acuity (Bailey-Lovie logMAR chart), and an ocular examination (slit lamp examination and dilated fundus examination) to exclude individuals with any ocular pathology, presbyopia, eye movement disorder, binocular vision anomaly, or systemic illness. Current contact lens wearers were not included (as this may have induced accommodation and convergence). Participants corrected visual acuity (BCVA) had to be of at least 0.1 for distance and N6 for near vision. They also had to have age-normative accommodation and vergence measurements. A total of 34 participants met the inclusion criteria and were included in the study.

### Mode of illumination

The study was conducted in accordance with established ergonomic guidelines, ensuring adherence to proper ergonomic standards. The ceiling was equipped with three 15-watt fluorescent bulbs, each of which could be controlled independently. This experiment examined three commonly employed illumination modes, and their brightness was measured using a lux meter. Mode A involved a viewer’s position illumination of 50 lux, achieved with a single fluorescent lamp. Mode B provided illumination of 100 lux at the viewer’s position, utilising two fluorescent lamps. Mode C employed three fluorescent lamps, illuminating 150 lux at the viewer’s position.

### Measurements

The study assessments included a cover test and a prism cover test (using a prism bar) to measure any deviation. Ocular motility was examined using the broad-H test, while the version was evaluated in nine specific eye positions. The push up test was utilised to determine the near point of accommodation monocularly (right and left) and with both eyes open. The measurement of the accommodative facility, expressed in cycles per minute, was conducted for both monocular and binocular viewing. Test targets of short, three-letter words in N8 font size were employed when assessing the lead and lag of accommodation, using the monocular estimated method. A typical lag was considered to fall between +0.25 and +0.75D (Scheiman & Wick 2014).

Near point of convergence was measured using an accommodative target. The measurement of fusional vergence amplitude (prism dioptres), both for distance and near vision, was conducted using a prism bar with step vergence. The evaluation included both negative fusional vergence and PFV. The normative values and diagnostic standards set by Scheiman and Wick were used as a reference (Scheiman & Wick 2014).

**Figure 1:**
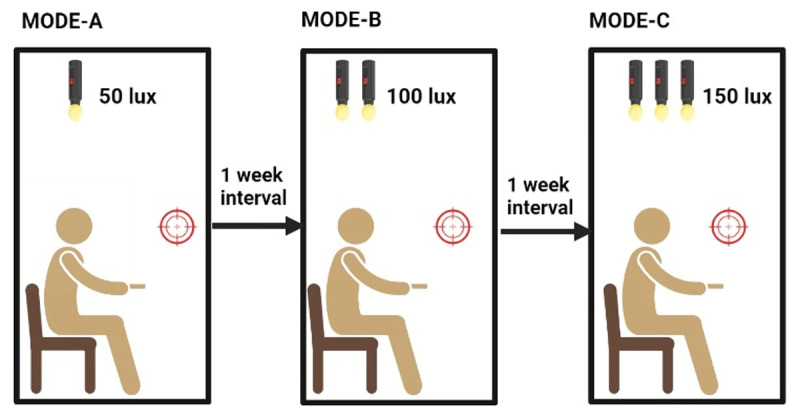
Demonstration of experimental sequence.

To prevent eye strain and fatigue, PFV was measured first. This was prioritised over the measurement of pupil diameter and contrast sensitivity because their measurements required less illumination and more convergence, potentially impacting the results. The study involved three different illumination conditions, with each subject being tested after a one-week interval. In the first week of testing, PFV, contrast sensitivity, and pupil diameter were measured under 50 lux lighting. Following a one-week break, the same procedures were repeated under 100 lux lighting. Finally, after one week of testing under 100 lux, the identical procedures were repeated, this time under 150 lux lighting conditions (see Figure 1). Contrast sensitivity threshold (Log CS) was measured using a Pelli Robson contrast sensitivity chart. A ruler was used to measure pupil diameter.

### Statistical analysis

Microsoft Excel 365 and SPSS (version 27.0.0) were used for descriptive and inferential date statistics respectively. The normality of the data was assessed using the Shapiro-Wilk test, revealing that the data was not normally distributed. Friedman’s test was employed to compare the near PFV across all three illuminations. Similarly, Friedman’s test was used to compare PFV, contrast sensitivity, and pupil diameter across the three illumination environments. The Wilcoxon signed rank test was used to check the comparison of near PFV between two different levels of illumination (50 and 100, 100 and 150, 50 and 150 lux).

## Results

Thirty-four participants who met the inclusion criteria, completed the study over three weeks. The mean age group of participants was 23.08 ± 1.40 years. This study included 15 males (46%) and 19 females (56%).

**Figure 2:**
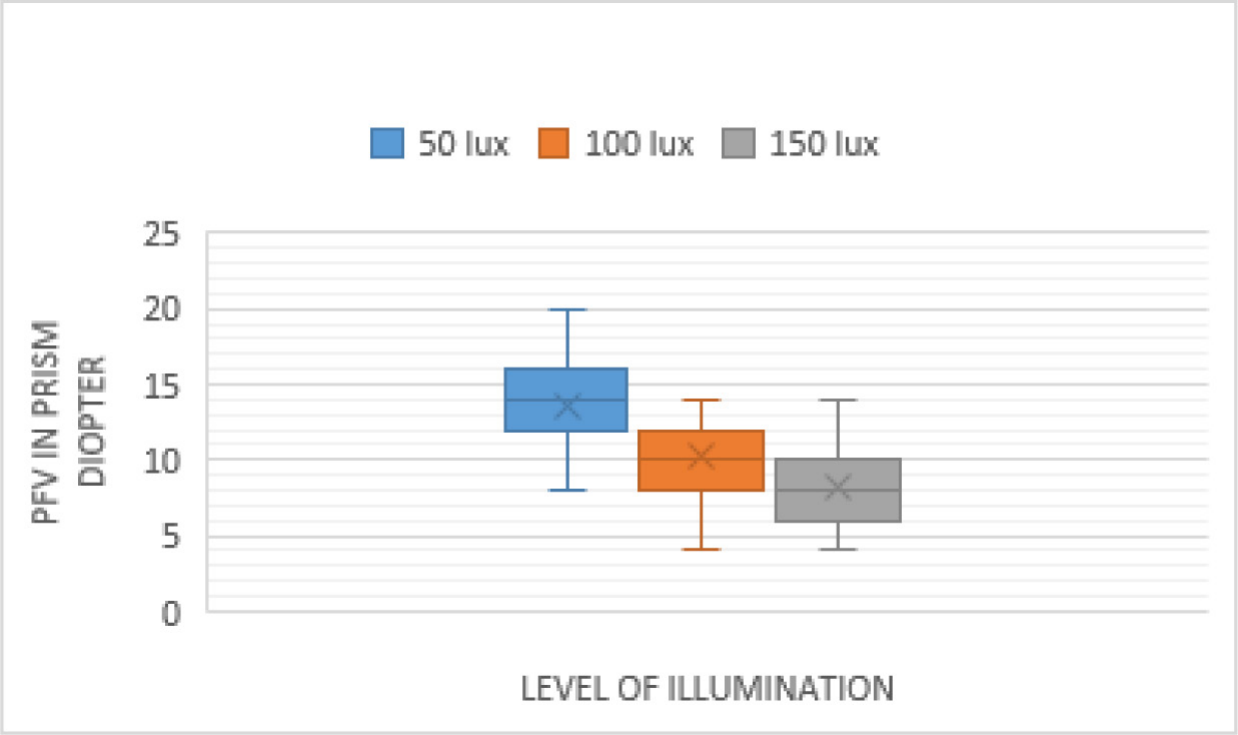
Comparison of near PFV—Blur point in three different Illuminations.

**Figure 3:**
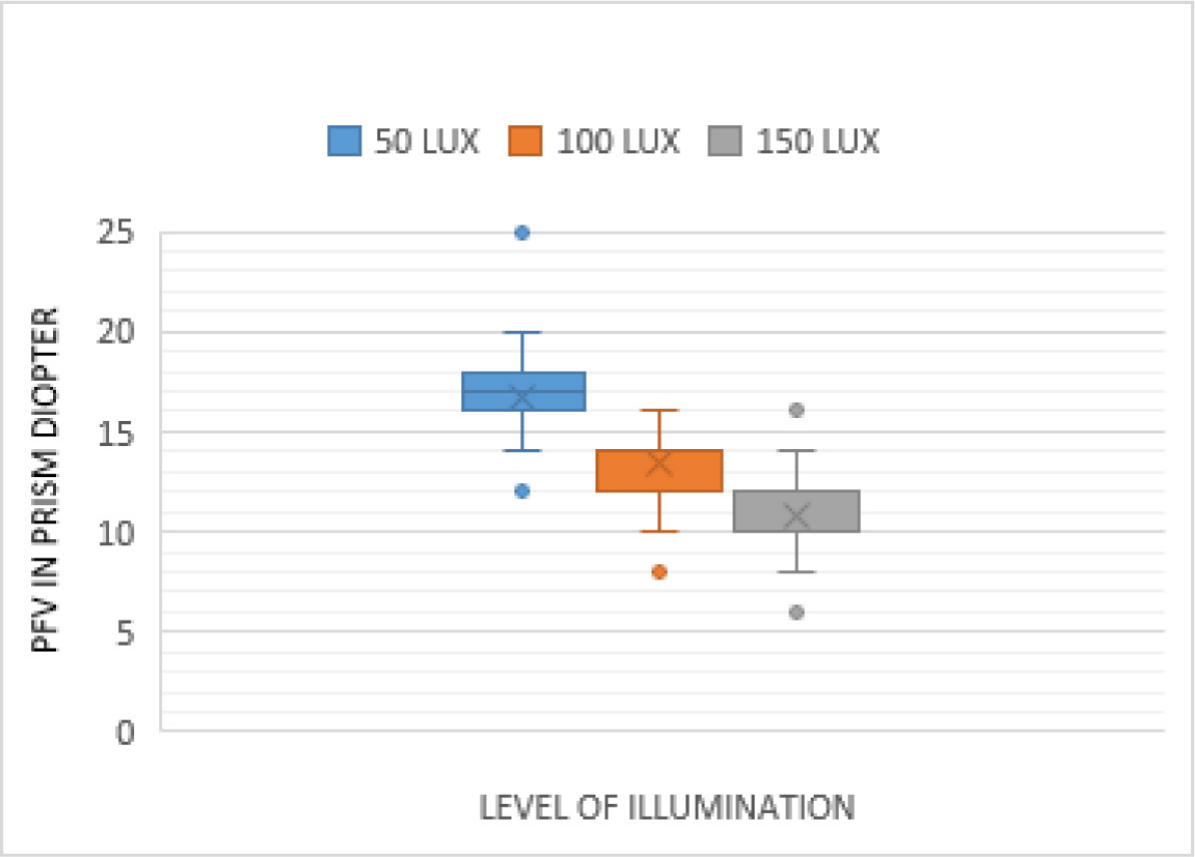
Comparison of near PFV—Break point in three different illuminations.

**Figure 4:**
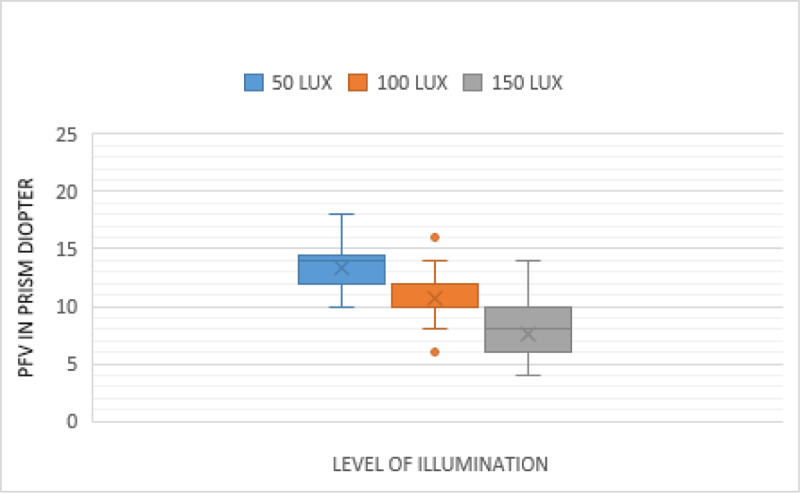
Comparison of near PFV—Recovery point in three different illuminations.

The measurements of PFV at near in the different levels of illumination are shown in Figure 2 (blur point), Figure 3 (break point), and Figure 4 (recovery point). As illumination increased, the PFV measurement decreased for the blur, break, and recovery points. There was a statistically significant effect of illumination on near PFV: blur (*p* < 0.001), break (*p* < 0.001), and recovery (*p* < 0.001) points (Friedman test) (Table 1). The difference in the PFV at near, between each level of illumination, was significant for the blur, break, and recovery points (Wilcoxon signed rank test) (Table 2).

**Table 1:** Comparison of near PFV in different levels of illumination (Friedman test).


NEAR-POSITIVE FUSIONAL VERGENCE	ILLUMINATION						*P* VALUE

	LOW (50 lux)		MEDIUM (100 lux)		HIGH (150 lux)		
		
	MEAN (PD)	SD	MEAN (PD)	SD	MEAN (PD)	SD	

**Blur point**	13.52	±2.51	10.23	±2.29	8.23	±2.18	<0.001

**Break point**	16.73	±2.4	13.47	±1.86	10.82	±2.26	<0.001

**Recovery point**	13.35	±2.18	10.7	±2.13	7.58	±2.73	<0.001


**Table 2:** Comparison of near PFV in different levels of illumination (Wilcoxon signed rank test).


ILLUMINATION (Lux)	POSITIVE FUSIONAL VERGENCE (NEAR)								

	BLUR POINT			BREAK POINT			RECOVERY POINT		
		
	MEAN	SD	*P* VALUE	MEAN	SD	*P* VALUE	MEAN	SD	*P* VALUE

50	13.52	±2.51	<0.001	16.73	±2.4	<0.001	13.35	±2.18	<0.001
		
100	10.23	±2.29		13.47	±1.86		10.7	±2.13	

100	10.23	±2.29	<0.001	13.47	±1.86	<0.001	10.7	±2.13	<0.001
		
150	8.29	±2.18		10.82	±2.26		7.58	±2.73	

50	13.52	±2.51	<0.001	16.73	±2.4	<0.001	13.35	±2.18	<0.001
		
150	8.29	±2.18		10.82	±2.26		7.58	±2.73	


Both monocular and binocular comparisons of contrast sensitivity with different illumination were studied. The mean (±SD) contrast sensitivity in 50 lux (right eye 1.80 ± 0.15, left eye 1.80 ± 0.15, both eyes 1.95 ± 0.15), 100 lux (right eye 1.80 ± 0.15, left eye 1.85 ± 0.15, both eyes 1.95 ± 0.15), 150 lux (right eye 1.95 ± 0.15, left eye 1.95 ± 0.15, both eyes 1.95 ± 0.15). There was no statistically significant difference between contrast sensitivity at different illumination levels (RE: *p* = 0.36, LE: *p* = 0.36, or BEO: *p* = 0.36). Pupil diameter measurements are shown in Table 3. As expected, as illumination increased, pupil diameter significantly decreased (Friedman test).

**Table 3:** Comparison of pupil diameter in different levels of illumination.


PUPIL DIAMETER	ILLUMINATION						*P* VALUE

	LOW (50 lux)		MEDIUM (100 lux)		HIGH (150 lux)		
		
	MEAN (mm)	SD	MEAN (mm)	SD	MEAN (mm)	SD	

Right eye	5.44	±0.61	4.17	±0.52	3.44	±0.5	<0.001

Left eye	5.44	±0.61	4.17	±0.52	3.44	±0.5	<0.001


## Discussion

The present study revealed a significant increase in near-positive fusional vergence (PFV) measurements under lower illumination levels, specifically in blur, break, and recovery points. As anticipated, the size of the pupils exhibited a notable increase under conditions of reduced illumination. There was no observed alteration in contrast sensitivity across varying levels of illumination.

Our findings are similar to the results of others, where increased PFV was measured as room illumination decreased (Jiang et al. 1991). PFV has also been found to change in different illumination while using a visual display unit (Majumder & Sinathamby 2017). When measuring accommodative and convergence demand during observation of a 3D object under two different lighting conditions, accommodative and convergence demands have also been found to be higher under low-light conditions compared to bright-light conditions (Okada et al. 2013).

Changes in near point of accommodation and near point of convergence have been noticed in different lighting conditions in terms of direct-indirect, direct, indirect, and compound (Wolska 2003). Jiang suggested that both vergence and accommodation tend to move toward new and individually characteristic resting postures when brightness is reduced for longer duration. An additional investigation determined that both the illumination mode and the utilisation of 3D viewing had an impact on ocular health (Jiang et al. 1991). This could be attributed to variations in luminance at the viewer’s position, the orientation of the light source, or the familiarity of the participants with their viewing surroundings (Chen et al. 2017). They suggested that front illumination is a favourable option for enhancing the visibility of 3D displays. Illumination source can also affect other ocular parameters, such as reading speed and visual performance. The reading rate was observed to be highest among males under compact fluorescent light, while females exhibited the fastest reading rate under fluorescent tube light (Ram & Bhardwaj 2018). The visual task setting parameters of white light and colour text were found to be more effective in average and low screen brightness conditions (Lin & Huang 2014). It was preferable to use blue as the font colour in low-luminance. This study examines the impact of varying levels of illuminance from LED lighting on users’ visual comfort and reading performance. Based on the guidelines provided by the Turkish Standards TS EN 12464, a lighting level of 1500 lux has been identified as optimal for reading tasks (Avci & Memikoğlu 2017).

Kim measured contrast sensitivity using an Arden Contrast Sensitivity System in an incandescent electric lamp and the influence of illumination on the contrast sensitivity function. The illumination intensities were set to 50, 100, 200, 500, and 1,000 lux. The contrast sensitivity function was saturated at 500 lux light in both monocular and binocular conditions (Kim & Kim 1987). In our current study, we observed no statistically significant variation in contrast sensitivity across varying levels of illumination, specifically at 50, 100, and 150 lux. The findings of the study imply a link between illumination type and visual performance in clinical settings, and showcase that illumination levels can affect PFV measurements. The present research supports the clinical need to provide advice on appropriate illumination.

We recognise the limitations of the study as we had a limited sample size (although this was calculated), and a limited number of illumination levels. We also recruited young adults who were asymptomatic and had good vision, which limited our ability to make conclusions about clinical populations or clinical scenarios. Future studies may include recruiting participants in different age groups, analysis of other vergence and accommodation parameters, and recruitment of clinical populations.

## Conclusion

The results of this study support that PFV measurements were affected by room illumination. Higher PFV values at near were measured in lower levels of illumination.

## Data Accessibility Statement

The data is available with examiner only for the research purposes.

## Figures and Tables

**Table:** 


CONTRIBUTION DETAILS	CONTRIBUTOR 1	CONTRIBUTOR 2	CONTRIBUTOR 3	CONTRIBUTOR 4

Concepts	ü	✓	✓	

Design	✓	✓	✓	✓

Definition of intellectual content		✓	✓	✓

Literature search	✓	✓	✓	

Clinical studies	✓	✓	✓	

Experimental studies	✓	✓	✓	

Data acquisition	✓	✓	✓	

Data analysis	✓	✓	✓	

Statistical analysis	✓	✓	✓	

Manuscript preparation	✓	✓	✓	✓

Manuscript editing	✓	✓	✓	✓

Manuscript review	✓	✓	✓	✓

Guarantor	✓	✓	✓	✓


## Appendix

The calculated sample size was 34.

Sample size calculation

Fisher & Law:

n = Sample size, Z = Standard normal variant = 1.96 (If we consider 95% confidence interval then Z score is 1.96.) P = Expected proportion in population based on previous studies or pilot studies (0.021) d = Absolute error or precision of 5% (0.05)

Sample size calculation = ((1.96)^2^ * 0.021 * (1–0.021))/(0.05)^2^ = 34
